# MiR-199a-5P promotes osteogenic differentiation of human stem cells from apical papilla *via* targeting IFIT2 in apical periodontitis

**DOI:** 10.3389/fimmu.2023.1149339

**Published:** 2023-03-30

**Authors:** Jing Hu, Xia Huang, Liwen Zheng, Yuxin Zhang, Huan Zeng, Li Nie, Xiaoxiao Pang, Hongmei Zhang

**Affiliations:** ^1^ Chongqing Key Laboratory for Oral Diseases and Biomedical Sciences, the Affiliated Hospital of Stomatology of Chongqing Medical University, Chongqing, China; ^2^ Department of Pediatric Dentistry, the Affiliated Stomatology Hospital, Chongqing Medical University, Chongqing, China; ^3^ Chongqing Municipal Key Laboratory of Oral Biomedical Engineering of Higher Education, College of Stomatology, Chongqing Medical University, Chongqing, China

**Keywords:** apical periodontitis, microRNA, miR-199a-5p, human stem cells from apical papilla (hSCAPs), osteogenic differentiation, bone regeneration

## Abstract

**Introduction:**

Periapical alveolar bone loss is the common consequence of apical periodontitis (AP) caused by persistent local inflammation around the apical area. Human stem cells from apical papilla (hSCAPs) play a crucial role in the restoration of bone lesions during AP. Studies have recently identified the critical role of microRNAs (miRNAs) involved in AP pathogenesis, but little is known about their function and potential molecular mechanism, especially in the osteogenesis of hSCAPs during AP. Here, we investigated the role of clinical sample-based specific miRNAs in the osteogenesis of hSCAPs.

**Methods:**

Differential expression of miRNAs were detected in the periapical tissues of normal and patients with AP via transcriptomic analysis, and the expression of miR-199a-5p was confirmed by qRT-PCR. Treatment of hSCAPs with miR-199a-5p mimics while loaded onto beta-tricalcium phosphate (β-TCP) ceramic particle scaffold to explore its effect on osteogenesis *in vivo*. RNA binding protein immunoprecipitation (RIP) and Luciferase reporter assay were conducted to identify the target gene of miR-199a-5p.

**Results:**

The expression of miR-199a-5p was decreased in the periapical tissues of AP patients, and miR-199a-5p mimics markedly enhanced cell proliferation and osteogenic differentiation of hSCAPs, while miR-199a-5p antagomir dramatically attenuated hSCAPs osteogenesis. Moreover, we identified and confirmed Interferon Induced Protein with Tetratricopeptide Repeats 2 (IFIT2) as a specific target of miR-199a-5p, and silencing endogenous IFIT2 expression alleviated the inhibitory effect of miR-199a-5p antagomir on the osteogenic differentiation of hSCAPs. Furthermore, miR-199a-5p mimics transfected hSCAPs loaded onto beta-tricalcium phosphate (β-TCP) scaffolds induced robust subcutaneous ectopic bone formation *in vivo*.

**Discussion:**

These results strengthen our understanding of predictors and facilitators of the key AP miRNAs (miR-199a-5p) in bone lesion repair under periapical inflammatory conditions. And the regulatory networks will be instrumental in exploring the underlying mechanisms of AP and lay the foundation for future regenerative medicine based on dental mesenchymal stem cells.

## 1 Introduction

Apical periodontitis (AP) is a common oral disease characterized by alveolar bone destruction and inflammatory disorder of periapical tissues, which often cause the severe arrest of root development, resulting in masticatory dysfunction and even loose or lost teeth that reduce the quality of life of individuals (1, 2). In the acute stages of AP, serous exudation, tissue edema, dilatation, and hyperemia of periodontal vascular are the main manifestations, while patients with AP in the chronic inflammatory stage usually exhibit a pathological condition with the formation of inflammatory granulation tissue in the apical area, finally leading to periapical bone destruction (3, 4). However, the exact mechanisms that contributed to these clinical and pathological manifestations remain unclear.

As a subgroup of dental mesenchymal stem cells (MSCs), human stem cells from apical papilla (hSCAPs) can be obtained from the apical tissue of underdeveloped permanent teeth and have been identified as promising seed cells in tissue engineering due to their self-renewal and multi-lineage differentiation potential (5–7). Owing to the capacity to diverge into distinct cell lineages, such as odontogenic, neurogenic, chondrogenic and osteogenic, hSCAPs play a vital role in the development of the root, pulp-dentin complex, and alveolar bone (8, 9). In particular, it has been reported that SCAPs may have superior osteogenic differentiation capacity compared to bone marrow mesenchymal stem cells (BMSCs) (10). Nonetheless, it was also reported that an inflammatory microenvironment could alter the hallmarks of SCAPs, leading to an inhibitory or increasing effect on osteogenic differentiation (11, 12). Effective osteogenic differentiation of MSCs is regulated by numerous factors, including physical, chemical, and biological factors, which may stimulate different signaling pathways, transcription factors, and microRNAs (miRNAs), to direct MSCs differentiated toward osteoblast lineage (13–16). However, the exact role of miRNAs in promoting osteogenic differentiation has yet to be fully understood.

MiRNAs are an evolutionarily conserved set of small non-coding RNAs of approximately 18-22 nucleotides, and display their functions mainly *via* binding to the 3′ untranslated regions leading to degradation or post-transcriptional repression of the mRNA targets (17). Studies have previously implicated the essential regulatory roles of miRNAs in diverse biological or pathological processes, including tumor metastasis, cellular differentiation, proliferation, apoptosis, and tissue development (18–20). The pivotal roles of miRNAs in osteogenesis, such as osteoblast differentiation, angiogenesis, and intra-chondral bone formation have also been identified (21–23). Additionally, previous studies have revealed that miRNAs may play crucial roles in the development and progression of oral diseases, such as apical periodontitis, pulpitis and periodontitis (24–27). However, among more than 2,000 miRNAs identified in humans, only a few were reportedly involved in apical periodontitis (28–30) although their role in regulating osteogenesis of hSCAPs and in clinical apical periodontitis samples have not been validated. Therefore, it is of significance to determine the important roles of miRNAs in promoting osteogenesis in inflammatory periapical tissues as this line of investigation is essential to expanding our current endodontics knowledge and exploring new treatment strategies.

Here, to explore the role of miRNAs in the osteogenesis of hSCAPs during AP, we conducted high-throughput microRNA RNA-seq and validation in extensive clinical samples. We found a novel profile of miRNA in periapical tissues with AP patients, which is helpful to identify the impact of miRNAs which exert significant predictors and facilitators of bone lesion repair in apical periodontitis. Importantly, we demonstrated that miR-199a-5p effectively promoted the osteogenic activity of hSCAPs both *in vitro* and *in vivo via* directly regulating *IFIT2* expression, which suggests its possibility to be potentially utilized to facilitate bone regeneration during apical periodontitis.

## 2 Materials and methods

### 2.1 Collection and high-throughput RNA-Seq analysis of periapical tissue samples

The use of patient samples was approved by the Ethics Committee of the Affiliated Stomatological Hospital of Chongqing Medical University. Samples of periapical tissue were acquired from each patient with informed written consent. All relevant procedures were performed following the approved guidelines. As is recommended by the clinical guidelines for endodontics, the diagnosis of apical periodontitis is based on the history, clinical examination, and periapical radiographic images (31). According to the correct clinical diagnosis, the periapical tissue samples were obtained from patients with severe periapical infection requiring extraction and whose roots had not yet been subjected to physiological resorption. The health control tissue samples were obtained from retained deciduous anterior teeth with a remaining root length greater than 2/3 and without carious. Furthermore, non-peer patients with systemic diseases such as diabetes, heart disease, asthma, etc., and with root resorption greater than 1/3 were excluded. A specific list of the inclusion and exclusion criteria for apical periodontitis is described in **Table S1**. The extracted teeth were placed in pre-cooled RNALater™ reagent (Beyotime, Shanghai, China) immediately and then rinse with cooling phosphate-buffered saline (PBS, Hyclone, UT, USA), the periapical tissue was quickly scraped (complete within 5 min on ice) and chilled in liquid nitrogen for 15 min, then kept at -80°C.

Periapical tissues from AP and healthy controls were subjected to high-throughput RNA-seq analysis by BMKCloud Biotechnology (Wuhan, China) to screen for differentially expressed miRNAs and mRNAs. Additionally, due to the small amount of periapical tissue in healthy controls, tissues from three different participants were pooled in each sample for sequencing. Potential target genes of miRNA were also predicted by bioinformatics analysis of the ENCORI database (https://starbase.sysu.edu.cn/), and relevant pathway enrichment analysis was performed by the DAVID website.

### 2.2 hSCAPs isolation, identification, and osteogenic differentiation

The apical papillae of teeth with underdeveloped roots were gently separated according to approved guidelines by the Stomatological Hospital Affiliated with Chongqing Medical University. The papilla tissues were digested with type I collagenase (Sigma, MO, USA) solution, and then maintained in Dulbecco’s Modified Eagle’s Medium with low glucose (L-DMEM, Hyclone, UT, USA) containing 10% fetal bovine serum (FBS, Hyclone, UT, USA), 1% penicillin-streptomycin at 37°C with 5% CO2, and change the medium at 3-day intervals. Three passages of hSCAPs were used in subsequent experiments.

Surface markers of hSCAPs were analyzed by flow cytometry on a BD Accuri C6 flow cytometer (BD Biosciences, CA, USA). Briefly, Cells were stained with FITC rabbit anti-CD90 (Sino-Biological, Beijing, China), anti-CD29 (Sino-Biological, Beijing, China), and anti-CD45 (Sino-Biological, Beijing, China). FlowJoTM software (Tree Star, Inc., Ashland, OR, USA) was applied to analyze the results with statistical calculations of the percentage of positive cells for visualization in histograms.

For osteogenic differentiation induction, hSCAPs were incubated in an osteogenic medium containing L-DMEM with 10% FBS, 100nM dexamethasone (Sigma, MO, USA), 10 mM β-glycerophosphate (Sigma, MO, USA), and 50 ug/ml ascorbic acid (Sigma, MO, USA).

### 2.3 Transient transfection of miRNA mimic, antagomir, NC, and siRNA

The miRNA mimics, antagomir and siRNAs were generated by Tsingke Biotechnology Co., Ltd. (Beijing, China), and then they were transfected into hSCAPs with HiPerFect transfection reagents (QIAGEN, Duesseldorf, Germany) according to the producer’s instructions. MiRNA mimics and NC were transfected at a concentration of 20 nM, while miR-199a-5p antagomir was used at 50 nM and incubated for 24 h. And then, the transfection medium was replaced with a normal growth medium or osteogenic induction medium to terminate the transfection according to the experimental needs. The specific sequences of miR-199a-5p mimics and antagomir are shown in **Table S2**.

### 2.4 Alkaline phosphatase assays and alizarin red S staining

ALP staining and ALP activity quantification assays were conducted after osteogenic induction at 3 or 7 days according to the instructions of an NBT/BCIP staining kit (Beyotime, Shanghai, China) and Alkaline Phosphatase Assay Kit (Nanjing Jiancheng Bioengineering Institute, China), respectively.

Alizarin red S staining was performed to detect mineralized nodules. Briefly, hSCAPs were first rinsed with PBS, fixed in 4% paraformaldehyde (Solarbio, Beijing, China), and subsequently dyed in 1% alizarin red solution (Solarbio, Beijing, China). For semi-quantitative analysis, Image J software will be utilized to analyze the ARs stained images, calculating the percentage of positive areas, three different stained images of ARs will be included in each group.

### 2.5 Total RNA extraction, reverse transcription, and quantitative real-time polymerase chain reaction

The RNAeasy™ Plus Animal RNA Isolation Kit (Beyotime, Shanghai, China) was utilized to extract total RNA. Reverse transcription was performed by using random hexamer primers or miRNA-specific stem-loop RT primers followed by the PrimeScript^®^ RT reagent kit instructions (TaKaRa, Tokyo, Japan), and cDNA generated from mRNA or miRNA are used as templates for amplified with TB Green Premix Ex Taq II (TaKaRa, Tokyo, Japan). In addition, Poly(A) was added to the 3’ end of the miRNAs, followed by a reverse transcription reaction with Oligo(dT)-Universal Tag reverse transcription primers to generate the first strand of cDNA corresponding to the miRNA and then measured by qPCR with specific forward primers and commercially accessible reverse primers according to the producer’s instructions (TIANGEN, Beijing, China). The relative expression of mRNA or miRNA was assessed by standardizing with those of GAPDH or U6, respectively. The primer sequences which were used in this research are shown in **Tables S3**, **S4**.

### 2.6 CCK-8 assay

Approximately 3×10^3^ cells/well were incubated in a 96-well plate and subsequently transfected with miR-199a-5p mimics, antagomir, NC, and siRNA. After transfection, replaced the original medium with a fresh complete medium containing 10 μL CCK8 reagent at specific time points, and incubated with cells for 1 h, then measure the absorbance at 450nm in a microplate reader (Perkin Elmer, Waltham, USA) and experiments were carried out in triplicates.

### 2.7 Crystal violet staining assay

Seed the hSCAPs in 35-mm dishes and after the cells are plastered, transfected with NC, miR-199a-5p antagomir, and mimics. Crystal violet (Beyotime, Shanghai, China) staining assays were conducted on these transfected cells at different indicated time points following the reagent instructions. Next, dissolve the stained cells in 33% acetic acid at room temperature, and measure the OD value at 570-590 nm for quantitative measurements.

### 2.8 RNA immunoprecipitation and RNA sequencing

RIP was performed with the Imprint^®^ RNA immunoprecipitation Kit (Sigma, MO, USA). Ten million cells were harvested and lysed in mild lysis buffer (B0314, Sigma, MO, USA) with protease inhibitors and ribonuclease inhibitors, and 5% of each cell lysate was removed as input. Protein A magnetic Beads (B0689, Sigma, MO, USA) were pre-incubated with anti-IgG (I5006, Sigma, MO, USA) or anti-AGO2 (ac186733, Abcam, UK) at room temperature with rotation for 30 min, and then cell lysate was added for further incubation with rotation overnight at 4 °C. The precipitated RNA was extracted by using an RNA Isolation Kit with Spin Column (Beyotime, Shanghai, China) following the producer’s instructions.

The rRNA was removed from the immunoprecipitated RNA, and then the products were subjected to high-throughput sequencing by Huada (BGI) Medical Laboratory Co., LTD (Wuhan, China). Filter the sequencing data with SOAPnuke26 (32) to obtain clean data, which were then stored and mapped to the reference genome using HISAT2. Dr. Tom’s multi-omics data mining system (https://biosys.bgi.com) was then applied to conduct data mining and analysis.

### 2.9 Western blot

Extraction of the total protein from cells in RIPA lysis buffer (Beyotime, Shanghai, China) containing the cocktail. Approximately 25 μg of protein was detached *via* a 10% sodium dodecyl sulfate-polyacrylamide electrophoresis (SDS-PAGE) gel, and further transmitted to 0.45 µm PVDF membranes (Millipore, MA, USA) and blocked in TBST containing 5% fat-free milk for about 1 hour. Later, incubate the membranes with antibodies. Visualization of immunological assays was performed by using a chemiluminescent ECL reagent (Beyotime, Shanghai, China). Primary antibodies were used as follows: anti-AGO2 antibody (Abcam, Cambridge, UK), anti-IFIT2 antibody (Proteintech, Wuhan, China), anti-RUNX2 antibody (Abcam, Cambridge, UK), anti-ALP antibody (Abcam, Cambridge, UK), anti-OPN antibody (Abcam, Cambridge, UK), and anti-GAPDH antibody (ZenBioscience, Chengdu, China).

### 2.10 Dual luciferase reporter analysis

293T cells are used for reporter gene assay. A wild-type reporter vector (IFIT2-3′-UTR- WT) was produced by fusing the IFIT2 3′-UTR sequence with the binding site of miR-199a-5p to the pmirGLO luciferase reporter vector (Promega). In addition, the mutant reporter vector (IFIT2-3′-UTR-MUT) was derived by inserting sentinel mutagenesis of the miR-199a-5p binding site from the IFIT2 3′-UTR sequence into the luciferase reporter vector. These reporter vectors were then cotransfected with miR-199a-5p mimics along with 293T cells by using Hieff TransTM Liposomal Transfection Reagent (YEASEN, Shanghai, China). Eventually, a dual luciferase reporter system (YEASEN, Shanghai, China) was applied to determine the luciferase activity 24 hours post-transfection. Light intensities were standardized with renilla luciferase.

### 2.11 *In vivo* ectopic bone formation and histological evaluation

All animal experiments were conducted under the ethical committee guidelines of the Affiliated Stomatological Hospital of Chongqing Medical University. Cells from each group (approximately 3×10^6^ cells per group) were loaded on *β*-tricalcium phosphate (*β*-TCP) porcelain granules (Bio-lu Biomaterials, Shanghai, China) and subcutaneously implanted the mixture into the flanks of 6-week-old BALB/c nude mice. 8 weeks later, these implants were obtained, fixed with paraformaldehyde (4%), decalcified in EDTA decalcification solution (Servicebio, Wuhan, China), and embedded in paraffin. Tissues embedded were serially sliced (5 μm) and processed for hematoxylin and eosin (H&E; Solarbio, Beijing, China) staining and Masson trichrome (Solarbio, Beijing, China) staining. Furthermore, the immunohistochemistry (IHC) staining was also carried out with anti-OCN antibodies (Abcam, Cambridge, UK) as previously described (33). The images were acquired by digital section scanner VS200 (Olympus, Japan).

### 2.12 SEM imaging and energy dispersive spectrometry analysis

The surface morphologies of β-TCP were observed and imaged by scanning electron microscopy (SEM, ZEISS, Sigma 300, Germany), and the particle sizes were evaluated by Nano Measurer 1.2 software based on its morphology map. An energy dispersive X-ray spectrometer (EDS, Oxford Instruments, Xplore, UK) was used to detect the elemental composition and distribution of the scaffolds.

### 2.13 Micro-CT analysis

Micro-CT scans were undertaken by Chongqing Key Laboratory of Oral Diseases and Biology. Micro-CT images were processed by Mimics Research 21.0 and 3 Matic Research 13.0 software to conduct 3D reconstruction and volumetric quantification. The eligible areas within the scaffold were picked to measure total volume (CT threshold above 4000HU) and bone volume (CT threshold between 4000HU-5500HU), for calculating the percentage of occupation of ectopic osteogenesis.

### 2.14 Statistical analysis

The studies were conducted independently three times at least, and differences in variables between groups were assessed with Graphpad 8.0 software using a student t-test or one-way ANOVA. *p* < 0.05 is regarded as statistically significant. Data were presented as the mean ± SD.

## 3 Results

### 3.1 Differential miRNAs expression identified by high-throughput microRNA RNA-seq analysis of periapical tissue isolated from teeth with normal and chronic apical periodontitis

**Figure 1:**
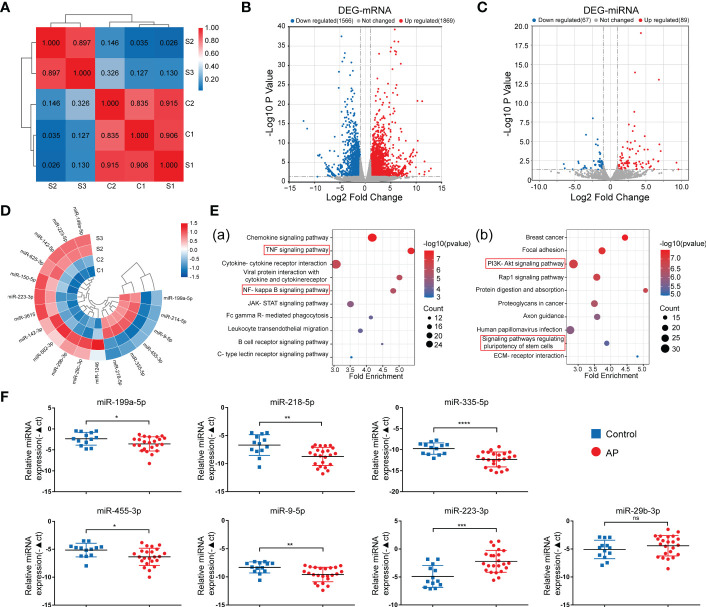
Identification of DE-miRNAs in periapical tissue. **(A)** Correlation analysis of the sequenced samples. **(B)** Volcano diagram of DE-mRNAs. **(C)** Volcano diagram of DE-miRNAs. **(D)** Circular heatmap of 12 up-regulated and 6 down-regulated miRNAs. **(E)** KEGG pathway enrichment analysis of the intersection genes between the DE-mRNAs and the predicted target genes of DE-miRNA. **(F)** Relative expression of the seven miRNAs in samples of periapical tissues (AP/Control:23/13). Data were presented as the mean ± SD. *p < 0.05, **p < 0.01, ***p < 0.001, and ****p < 0.0001, ns, no significance.

To explore the role of miRNA in the osteogenic differentiation of inflamed periapical tissue, conventional RNA-seq and microRNA RNA-seq were conducted to determine the distinct expression patterns of miRNA and mRNA between periapical tissues from AP patients (S) and health controls (C). Based on the correlation analysis between AP and control groups (**Figure 1A**), two samples from each group were selected to be further analyzed. Differential expression analysis based on |log2FC| (fold change) >1 and *p*-value<0.05 identified 1869 up-regulated mRNAs and 1566 down-regulated mRNAs (**Figure 1B**), as well as 89 up-regulated miRNAs and 67 down-regulated miRNAs. (**Figure 1C**). Then, with further parameters setting the basal expression level (counts>10), 12 up-regulated and 6 down-regulated miRNAs were detected in AP tissues compared to healthy controls, including miR-335-5p and miR-455-3p (**Figure 1D**), which were previously reported to enhance osteogenic differentiation of MSCs (34, 35), and were chosen for subsequent bioinformatics analyses.

First, *via* intersecting the target genes of the 18 DE-miRNAs predicted by databases with the DE-mRNAs identified by RNA-seq in our study, we found 445 up-regulated mRNAs and 633 down-regulated mRNAs, respectively (**Figures S1A, B**). Second, Kyoto Encyclopedia of Genes and Genomes (KEGG) pathway enrichment analysis was used to classify potential functions of these genes (**Figure 1E**), and we found that the majority of enriched pathways were pointed to the classic inflammation-related signaling pathways, such as TNF and NF-KB signaling pathways (36–38), or pathways regulating stem cell pluripotency and PI3K-Akt signaling pathways associated with osteogenic differentiation (39). By exploring the upstream miRNAs of the genes included in these enriched pathways, seven miRNAs, including upregulated miR-29b-3p and miR-223-3p, as well as down-regulated miR-335-5p, miR-9-5p, miR-218-5p, miR-455-3p, and miR-199a-5p were identified, indicating that these differentially expressed miRNAs may exert osteogenesis effects in apical periodontitis. Lastly, the expression of these miRNAs was validated by qPCR analysis of the periapical tissues from 23 AP patients and 13 healthy controls (**Figure 1F**). Consequently, the above seven miRNAs were chosen as candidates for further study.

### 3.2 7 Validation of expression pattern of the seven differentially expressed miRNAs during osteogenic differentiation of hSCAPs

**Figure 2:**
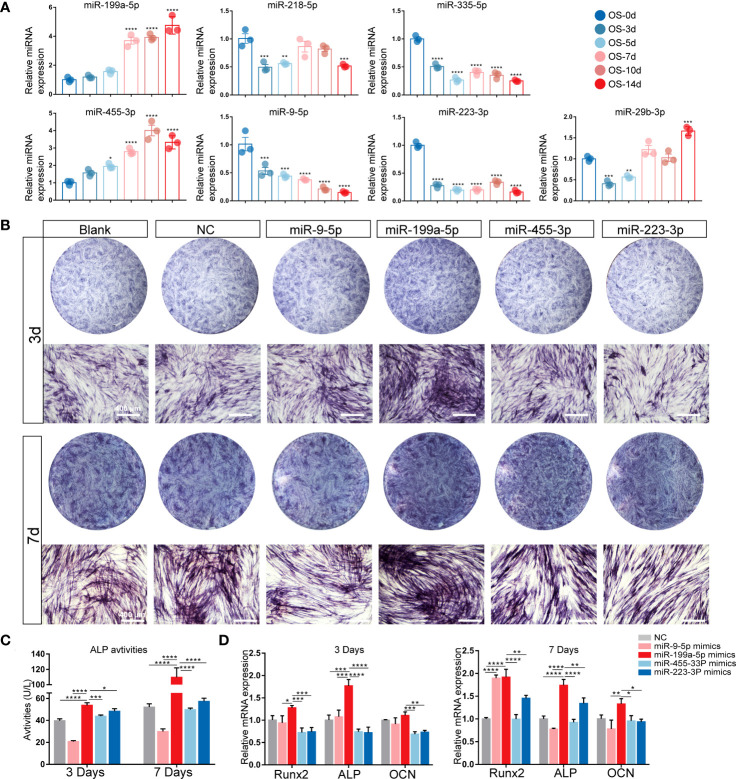
Expression patterns of the candidate miRNAs during osteogenic differentiation of hSCAPs. **(A)** Relative expression of the 7 screened miRNAs. **(B)** ALP staining of different miRNA overexpression groups after 3 and 7 days of osteogenic induction (scale bar = 400 μm). **(C)** ALP activity in the different groups. **(D)** The relative mRNA expression level of osteogenic marker genes RUNX2, ALP, and OCN. The data were shown as mean ± SD. *p < 0.05, **p < 0.01, ***p < 0.001, and ****p < 0.0001.

Since the target genes of 7 miRNAs were most enriched in the osteogenic-related signaling pathways, we isolated hSCAPs from young permanent teeth with underdeveloped roots first (**Figures S2A, B**). The expressions of surface markers were detected by FACS, and results showed that MSC surface markers CD90 and CD29 were positive in hSCAPs but negative for hematopoietic stem cell marker CD45 (**Figure S2C**). We also detected the expression levels of these candidate miRNAs at different time points of osteogenic differentiation of hSCAPs (**Figure 2A**) and found that the expression of miR-199a-5p and miR-455-3P gradually elevated, while miR-9-5p, miR-335-5p, and miR-223-3p expression gradually decreased during the osteogenic differentiation of hSCAPs.

We further compared the miRNA expression profiles in hSCAPs with that in periapical tissues to determine the candidate miRNAs involved in periapical osteogenesis. Four miRNAs, including miR-199a-5p, miR-9-5P, miR-455-3P and miR-223-3P, were selected and validated by early osteogenic phenotype, Alkaline phosphatase (ALP) staining. As demonstrated by ALP staining and ALP activity assay, the osteogenic differentiation of hSCAPs was significantly promoted by miR-199a-5p mimics (**Figures 2B, C**), which was more pronounced than other miRNA overexpression groups. Furthermore, only the miR-199a-5p overexpression group significantly upregulated the expression of osteogenic marker genes *RUNX2*, *ALP*, and *OCN* in hSCAPs (**Figure 2D**). Collectively, these data suggest that miR-199a-5p may modulate the osteogenesis of hSCAPs.

### 3.3 miR-199a-5p positively modulates the proliferation and osteoblast differentiation of hSCAPs

**Figure 3:**
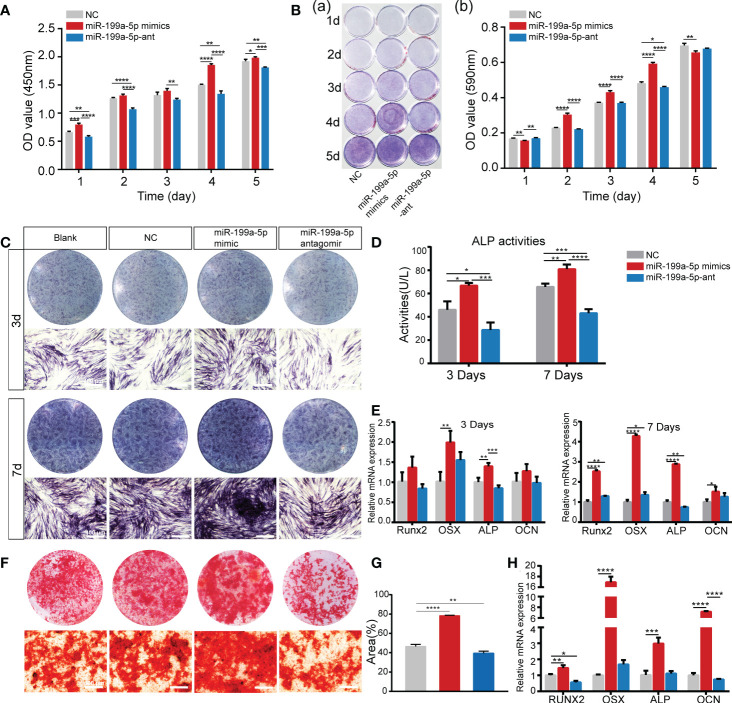
miR-199a-5p positively regulates the proliferation and osteogenic differentiation of hSCAPs *in vitro*. **(A)** The CCK8 assay. **(B)** The Crystal violet staining and quantification assay. **(C)** ALP staining of different groups after 3 and 7 days of osteogenic induction (scale bar = 400 μm). **(D)** ALP activity assay. **(E)** The relative mRNA expression level of osteogenic marker genes RUNX2, OSX, ALP, and OCN. **(F)** ARs staining assay (scale bar = 400 μm). **(G)** Semi-quantitative analysis of ARs stained images. **(H)** The relative mRNA expression level of osteogenic marker genes RUNX2, OSX, ALP, and OCN. The data were shown as mean ± SD. *p < 0.05, **p < 0.01, ***p < 0.001, and ****p < 0.0001.

To confirm the biological function of miR-199a-5p in cell proliferation and osteogenic differentiation of hSCAPs, we first transfected hSCAPs with miR-199a-5p mimics, antagomir, and negative control (NC), respectively. We found that the accumulation level of mature miRNAs still maintained several hundred-fold increases after transfection of miRNA mimics into hSCAPs for several days (**Figure S3**). The CCK-8 assay revealed that hSCAPs transfected with miR-199a-5p mimics exhibited increased proliferation as compared to NC, while decreased when miR-199a-5p was knocked down in hSCAPs (**Figure 3A**). Crystal violet staining revealed statistically significant increased numbers of cells in the miR-199a-5p overexpression group than those transfected with NC or miR-199a-5p antagomir after seeding at the same initial density (**Figures 3B**). These results reveal that miR-199a-5p may promote the proliferation of hSCAPs and enhance their self-renewal capacity.

Furthermore, we assessed the function of miR-199a-5p in regulating the osteogenic differentiation of hSCAPs. The hSCAPs transfected with miR-199a-5p mimics, antagomirs, and NC for 24h were incubated in osteogenic induction media for 3 or 7 days. Both the expression and activity of ALP were markedly increased in the miR-199a-5p overexpression group (**Figures 3C, D**). Moreover, we determined the expression levels of the osteoblast-relevant markers by qPCR and found that *RUNX2, OSX, ALP, and OCN* expression were significantly increased in the miR-199a-5p overexpressing group on 3 and 7 days of osteogenic induction (**Figure 3E**). As expected, we observed a significant decrease in ALP staining and activity with inhibition of miR-199a-5p in hSCAPs. Meanwhile, miR-199a-5p knockdown in hSCAPs led to *RUNX2* and *ALP* inhibition, while *OSX* and *OCN* expression were not affected. Alizarin red staining assay and semi-quantitative analysis revealed that miR-199a-5p overexpression remarkably enhanced calcium nodule deposition (**Figures 3F, G**). The qPCR analysis showed that miR-199a-5p expressing hSCAPs exhibited a dramatically increased expression of *ALP*, *OCN*, OSX, and RUNX2 than those in the control cells (**Figure 3H**). Taking together, we demonstrate that miR-199a-5p may facilitate the osteogenic differentiation of hSCAPs *in vitro*.

### 3.4 miR-199a-5p directly targets IFIT2 in hSCAPs

**Figure 4:**
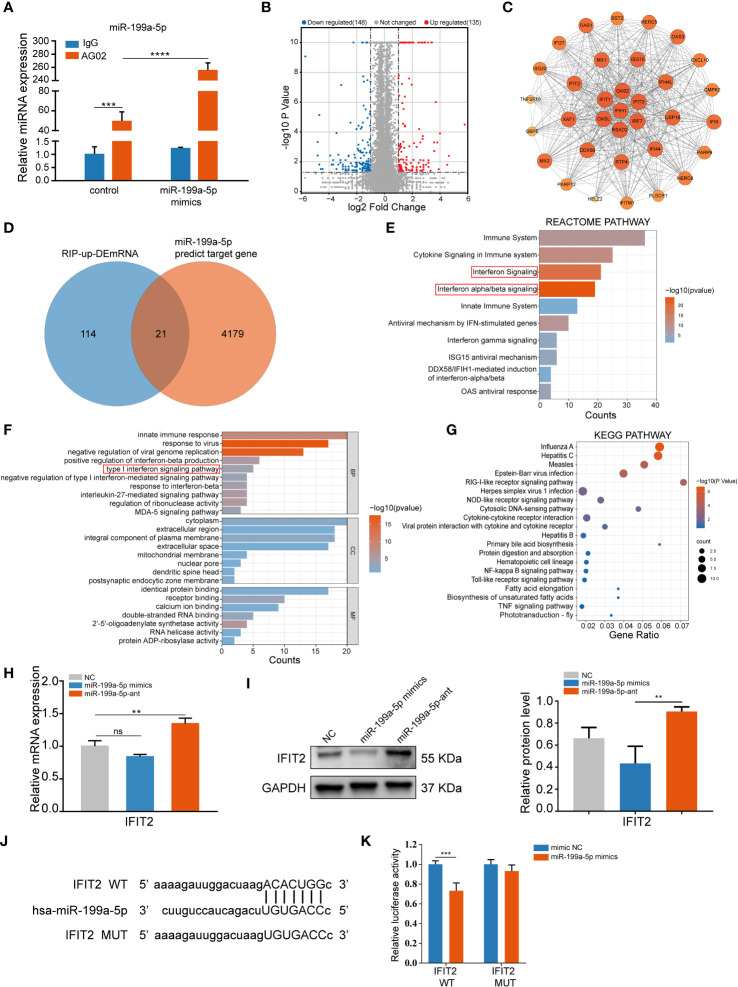
miR-199a-5p directly targets *IFIT2* in hSCAPs. **(A)** The relative expression level of miR-199a-5p in immunoprecipitates. **(B)** The volcano plot of differentially enriched mRNAs in the Ago2 immunoprecipitation complex between the miR-199a-5p overexpression and the control group. **(C)** Protein-Protein Interaction (PPI) analysis of the up-regulated genes. **(D)** Venn diagram of the intersection genes between the significantly up-regulated mRNAs (blue) and the predicted target genes of miR-199a-5p (orange). **(E)** Reactome pathway analysis of the up-regulated mRNAs. **(F)** GO analysis of the up-regulated mRNAs. **(G)** KEGG pathway enrichment analysis of the up-regulated mRNAs. **(H)** The relative expression level of IFIT2 at mRNA levels. **(I)** The protein expression levels of IFIT2. **(J)** Potential binding sites between lFIT2 and miR-199a-5p. **(K)** Dual-luciferase reporter assay. The data were shown as mean ± SD. **p < 0.01, ***p < 0.001, and ****p < 0.0001, ns, no significance.

To identify the target mRNAs post-transcriptionally modulated by miR-199a-5p involved in osteogenic differentiation of hSCAPs, we performed an Ago2 RIP-sequencing. Notably, it was determined that miR-199a-5p was enriched in the immunoprecipitates of the anti-AGO2 group when compared with the IgG group by qPCR, and miR-199a-5p enrichment was remarkably higher in the overexpression group than that in the controls (**Figure 4A**). Next, we performed RNA-seq analysis of differential expression genes enriched in AGO2 between the controls and the miR-199a-5p overexpression group. Based on *p*-value (<0.05), and fold change (>2), 135 up-regulated mRNAs were collected (**Figure 4B**), which were subsequently intersected with the predicted target genes of miR-199a-5p (**Figure 4D**). Consequently, 9 potential target genes were obtained and confirmed by qPCR in RIP products (**Figure S4A**). Among them, *IFIT2* was the most significantly elevated one.

Furthermore, Protein-Protein Interaction networks (PPI) analysis (**Figure 4C**) of the up-regulated genes in RIP-sequencing revealed multiple IFIT family genes that are closely linked and enriched (**Figure S4B**). Additionally, we conducted Gene Ontology (GO), Reactome pathway, and KEGG pathway enrichment analysis on these 135 significantly up-regulated genes, in which type 1 interferon signaling pathway was found to be involved (**Figures 4E-G**). Moreover, overexpression of miR-199a-5p in hSCAPs revealed a significant downregulation of IFIT2 expression at the protein level, while slightly declining expression of *IFIT2* at the mRNA level. Conversely, miR-199a-5p expression inhibition in hSCAPs enhanced IFIT2 expression at both mRNA and protein levels (**Figures 4H, I**). A luciferase reporter assay was conducted to confirm the direct association between the 3’UTR of IFIT2 and miR-199a-5p (**Figures 4J, K**). Collectively, these results demonstrate that miR-199a-5p binds directly to *IFIT2* and acts as a negative regulator of *IFIT2* in hSCAPs.

### 3.5 Knockdown of IFIT2 can rescue the impact of endogenous miR-199a-5p reduction on osteogenesis

**Figure 5:**
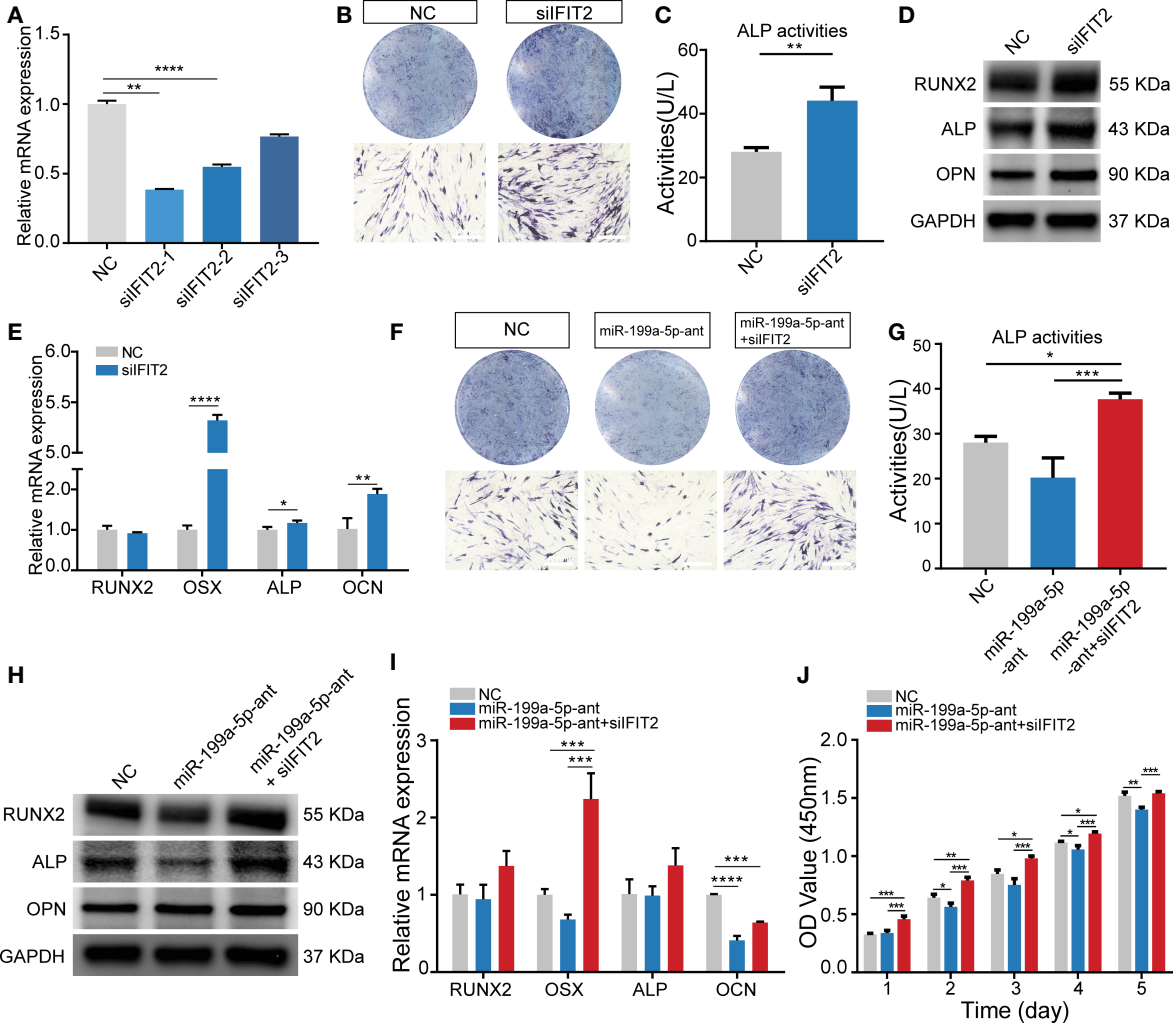
Knockdown of *IFIT2* can reverse the effect of endogenous miR-199a-5p reduction on osteogenesis. **(A)** The knockdown efficiency of the three pairs of IFIT2 siRNAs. **(B)** ALP staining after 3 days of osteogenic induction (scale bar = 400 μm). **(C)** ALP activity assays. **(D)** The protein expression levels of RUNX2, ALP, and OPN. **(E)** The relative mRNA expression level of osteogenic marker genes *RUNX2*, *OSX*, *ALP*, and *OCN*. **(F)** ALP staining after 3 days of osteogenic induction (scale bar = 400 μm). **(G)** ALP activity assay. **(H)** The protein expression levels of RUNX2, ALP, and OPN. **(I)** The relative mRNA expression level of osteogenic marker genes *RUNX2*, *OSX*, *ALP*, and *OCN*. **(J)** The CCK-8 assay. The data were shown as mean ± SD. *p < 0.05, **p < 0.01, ***p < 0.001, and ****p < 0.0001.

To investigate whether miR-199a-5p functionally targets *IFIT2* in modulating hSCAPs osteogenic differentiation, we first repressed *IFIT2* expression by transfecting hSCAPs with siRNAs against *IFIT2*. Three pairs of siRNA against *IFIT2* were tested and *IFIT2* expression was remarkably repressed by siIFIT2-1 at mRNA levels (**Figure 5A**). Consequently, siIFIT2-1 was chosen in subsequent functional experiments. By using *IFIT2*-specific siRNAs, the results revealed that the osteogenic differentiation of hSCAPs was upregulated after *IFIT2* knockdown as demonstrated by increased osteogenesis makers, including RUNX2, ALP, and OPN at the protein levels (**Figure 5D**) and *RUNX2*, *OSX, ALP*, and *OCN* at the mRNA levels (**Figure 5E**). Furthermore, ALP staining and quantification assay showed that *IFIT2* downregulation remarkably enhanced ALP activities of hSCAPs in the induction of osteogenesis (**Figures 5B, C**). Importantly, *IFIT2* silencing markedly reversed the reduced ALP activities induced by miR-199a-5p inhibition (**Figures 5F, G**). The altered expression of RUNX2, ALP at the protein levels and *RUNX2*, *OSX, ALP*, and *OCN* at the mRNA levels further reinforced a resemble rescue effect of IFIT2 inhibition. (**Figures 5H, I**). Since miR-199a-5p transfected hSCAPs showed increased proliferation, we conduct the CCK8 assay to determine if *IFIT2* knockdown could rescue the decreased proliferation of hSCAPs transfected with miR-199a-5p antagomir. The results showed that *IFIT2* silencing accelerated the proliferation of hSCAPs transfected with antagomir (**Figure 5J**). Taken together, the above results suggest that miR-199a-5p may regulate osteogenic differentiation of hSCAPs *via* targeting *IFIT2*.

### 3.6 *β*-TCP ceramic particles loaded with miR-199a-5p expression hSCAPs effectively promote bone formation *in vivo*

We conducted an ectopic osteogenesis assay in BALB/c nude mice to further investigate the impact of miR-199a-5p on bone formation *in vivo*. Briefly, hSCAPs were transfected with NC or miR-199a-5p mimics and cultured for two days in osteogenic induction media. The transfected hSCAPs were then collected and loaded onto *β*-tricalcium phosphate (*β*-TCP) scaffolds, which subsequently were implanted subcutaneously into the flanks of mice (n=3).

We first analyzed the characteristics of the scaffolds. SEM imaging (**Figure S5A**) showed the surface morphology of the scaffolds, and the diameter of the ceramic particles was measured to be approximately 1.48 μm (**Figure S5B**). Furthermore, the elemental analysis indicated that the scaffold was composed of calcium, phosphate, and oxygen (**Figure S5C**), which contented 38.20%, 19.56%, and 41.53% by weight, respectively (**Figure S5D**).

**Figure 6:**
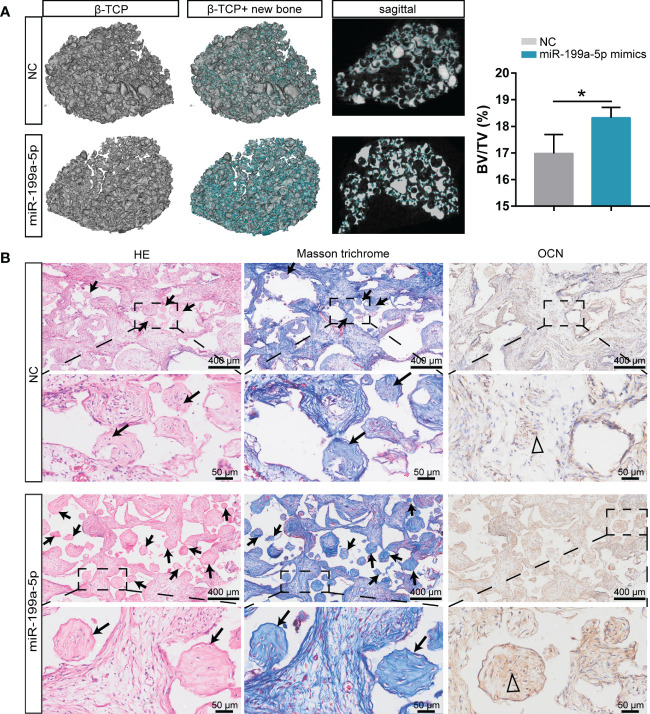
Overexpression of miR-199a-5p enhances hSCAPs osteogenic differentiation *in vivo*. **(A)** Left: Representative micro-CT images of 3D reconstruction and sagittal profiles of bone formation in *β*-TCP scaffolds of miR-199a-5p mimics (n = 3) and NC groups (n = 3). The blue areas: the region of new bone formation. Right: Quantitative analysis of BV/TV (%). The data were expressed as mean ± SD. *p < 0.05. **(B)** Representative images of H&E staining, Masson’s trichrome staining and immunohistochemical staining of OCN. The black arrows: bone formation. The triangle: positive cells.

The representative three-dimensional (3D) reconstruction and micro-CT images of sagittal profiles of the retrieved scaffolds revealed that the miR-199a-5p overexpression group had a much higher density of bone formation, as well as a greater BV/TV ratio (**Figure 6A**). H & E staining showed a significant increase of bone mass on scaffolds loaded with miR-199a-5p overexpressing hSCAPs and more collagenous tissue in Masson trichrome staining. Furthermore, immunohistochemistry analysis showed that the miR-199a-5p overexpression group had more OCN-positive cells in the bone fragments, as compared to the controls (**Figure 6B**). Taken together, these results indicate that miR-199a-5p overexpression promotes the osteogenesis of hSCAPs *in vivo* and *β*-TCP ceramic particles loaded with miR-199a-5p expressing hSCAPs display effective osteogenic capacity *in vivo*.

## 4 Discussion

In this study, to investigate the role of miRNAs during AP, microRNA and mRNA sequencing were performed on the periodical tissue samples from AP patients and healthy controls. By validation in extensive clinical samples and functional experiments, we identify a differentially expressed key miRNA (miR-199a-5p), which plays a critical role in the osteogenesis of hSCAPs during AP. We found that overexpression of miR-199a-5p could promote osteogenic differentiation of hSCAPs *in vivo* and *in vitro* by directly targeting IFIT2.

Inflammation of periapical tissues commonly presents with the destruction of the alveolar bone, which is generally treated by controlling the infection and eliminating the inflammation to promote the repair of the periapical bone defects (40–43). Currently, miRNAs, transglutaminases, and other agents have been found to be involved in the development and progression of oral diseases (25, 44, 45). Most importantly, miRNAs have been implicated as valid regulators that can modulate multiple biological processes, including bone regeneration and anti-inflammatory response (46, 47). As Shen and Silva (25) have reported that over 100 miRNAs were differentially expressed in AP and the highest expression of miR-10a-5p appeared to be involved in triggering anti-inflammatory signaling and promoting healing. It is noteworthy that different phases of inflammatory conditions may have different effects on the bone regenerative capacity of stem cells, as Liu et al. (2016) (48) showed that long-term exposure to pro-inflammatory cytokines inhibited the osteogenic differentiation of SCAPs. However, Hess et al. (2009) (49) revealed that TNF-α promoted osteogenic differentiation of human MSCs by triggering the NF-κB signaling pathway. The above results indicate that there are complex signals modulating the differentiation of stem cells under inflammatory conditions. Therefore, we further performed functional enrichment analysis and phenotypic validation of both up-regulated and down-regulated miRNAs-mRNAs networks to comprehensively identify key miRNAs that impact AP bone repair.

Previously, the role of miRNAs in apical periodontitis and pulp diseases has been explored by microarray studies, which identified several miRNAs that were significantly differentially expressed in AP, including miR-181 family, miR-10a-5p, and also miR-199a-5p (28, 30). However, the AP and control samples were not probed in the same chip, which decreased the reliabilities, while miR-199a-5p was not further validated in extensive clinical samples. Interestingly, in our study, we performed miRNA and mRNA sequencing of AP and control samples in the same batch, and we found that miR-199a-5p was one of the most differentially expressed miRNAs, which was further validated in adequate clinical samples to guarantee that the screened miRNAs display a stronger correlation with bone defect repair in AP. Meanwhile, we summarized the characteristics of the patients (**Figure S6A**) and conducted a correlation analysis between miR-199a-5p expression and periapical lesion status based on the periapical index (PAI) (**Figure S6B**), which is the most classic scoring system for the evaluation of AP (50). The results showed a negative correlation between miR-199a-5p expression and periapical lesion status (R=-0.5429) (**Figures S6C–E**). Subsequently, the functional analysis indicated that miR-199a-5p enhanced osteogenesis of hSCAPs, which may play a pivotal role in bone lesion repairment during AP. Besides, the pathway enrichment analysis of mRNA sequencing in clinical samples and AGO2-RIP sequencing in hSCAPs all pointed to various inflammatory pathways, including TNF signaling pathway and NF-kappa B signaling pathway, indicating that the miRNA identified in our study, miR-199a-5p, may exhibit great potency of promoting osteogenesis even in the scenario of inflammation, which needs to be further studied, such as the role of this key miRNA for bone defect repair under the inflammatory microenvironment and the impact of the immunological components, including inflammatory factors and biological responses on bone formation.

miRNAs are considered as ‘junk’ RNA to regulate most cellular events through identifying multiple target genes. A previous study has shown that miR-199a-5p overexpression could enhance osteoblast differentiation of human MSCs through regulation of the HIF1a-Twist1 pathway (51). However, we failed to find the genes enriched in AGO2-RIP samples involved in the HIF1a-Twist1 pathway in our study, which indicates that miR-199a-5p could facilitate osteogenic differentiations in different stem cells, but the specific mechanism may be different. Another aspect, miR-199a-5p was shown to positively regulate osteoclast differentiation by targeting Mafb protein (52), which suggests that miR-199a-5p may have a vital role in regulating bone homeostasis *via* simultaneously mediating osteoblast and osteoclast differentiation, but its specific role and underly mechanism needs to be further investigated.

IFIT2, belonging to the interferon-stimulated gene (ISG) family, is widely expressed in mammalian tissues, including bone marrow (53). Previous studies showed that the endogenous Interferon beta (IFN*β*, type-1 IFN) activity represses osteoblast differentiation *in vivo and in vitro* (54, 55) and that low levels of type I IFN-induced cellular IFN activity are commonly mirrored by ISG expression (56, 57). Interestingly, except for *IFIT2*, we found that *IFIT1*, *IFIT3*, and *IFI44L* were also enriched in AGO2 protein transfected with miR-199a-5p mimics, which strongly indicated that type-1 IFN response was blocked in the miR-199a-5p transfected hSCAPs, that may be the critical factor why miR-199a-5p promotes osteogenesis of hSCAPs, but the exact mechanism needs to be fully explored in the future. Additionally, overexpression of IFIT2 correlated with the diminished proliferative capacity of various cells (58, 59), which is consistent with our results that miR-199a-5p inhibiting *IFIT2* expression resulted in increased proliferation of hSCAPs. Collectively, these results reveal that miR-199a-5p promotes osteogenesis of hSCAPs *via* targeting *IFIT2* and blocking the endogenous type-1 IFN response.

For the regeneration of periapical bone tissue defects, SCAPs are valid candidates with excellent osteogenic capacity. According to the classical strategies for biomaterial substitutes used for bone tissue engineering (60), *β*-TCP ceramic particles were chosen as the scaffold, which displays excellent biocompatibility and osteoinductive capacities (61, 62) and is extensively used in clinical(63). Not surprisingly, *β*-TCP ceramic particles loaded with miR-199a-5p overexpressed hSCAPs exhibited more ectopic bone formation, which suggests that miR-199a-5p overexpressed hSCAPs hold potential for bone defects repairment and may be promising strategies for bone tissue regeneration. Furthermore, deploying an appropriate drug-delivery system for miR-199a-5p is needed to address its potential in orthotopic periapical tissue repair.

**Figure 7:**
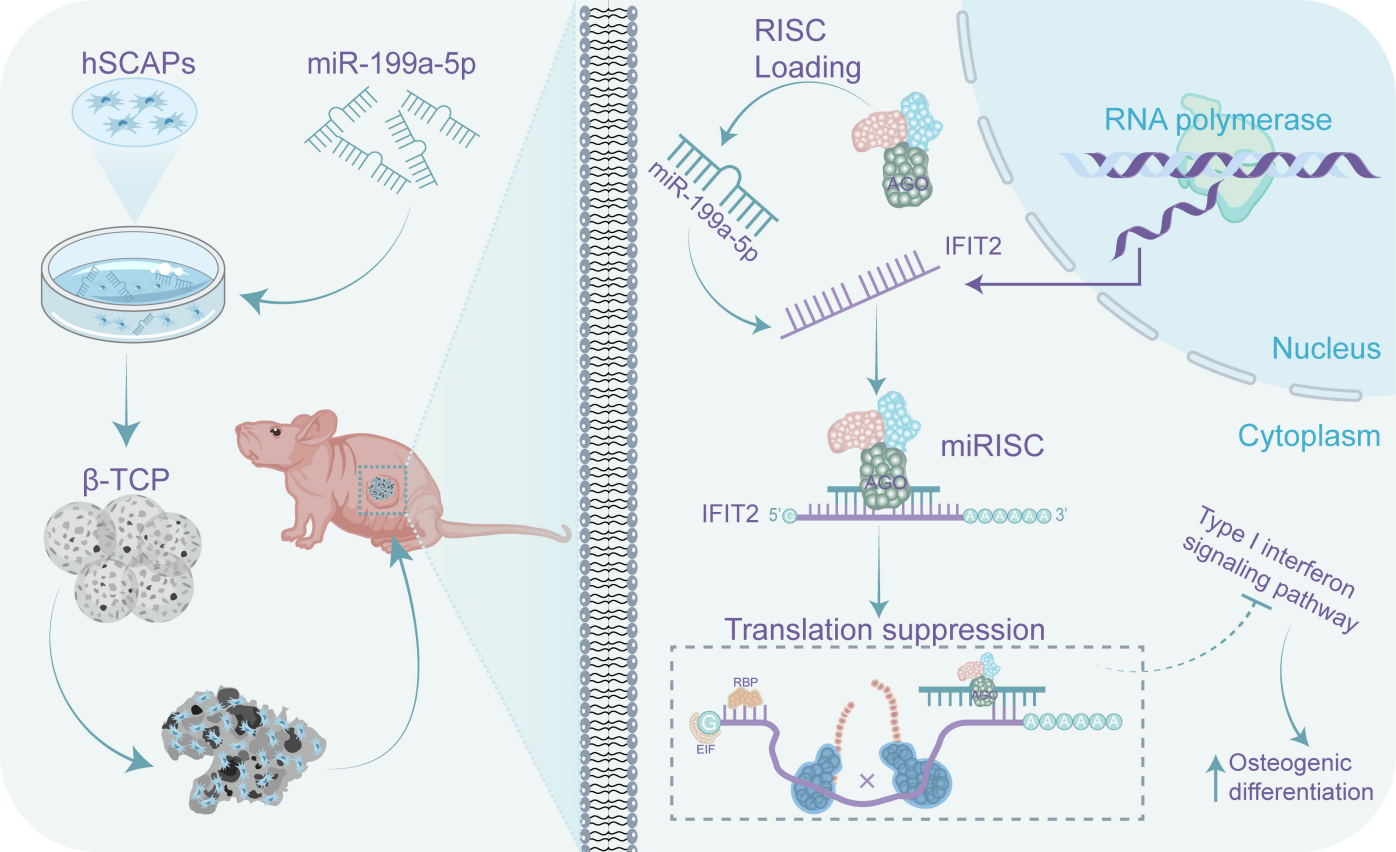
A schematic representation on how miR-199a-5p regulates the osteogenic differentiation of hSCAPs. miR-199a-5p promotes the osteogenic differentiation of hSCAPs *via* directly suppressing IFIT2 expression *in vitro* and *in vivo*, leading to blocking the type-1 interferon signaling.

In summary, in this study, we revealed the profile of miRNAs in periapical tissues of AP patients and healthy controls by miRNA sequencing. We found that the most differentially expressed key miRNA (miR-199a-5p) enhanced osteogenic differentiation of hSCAP *in vivo* and *in vitro* by targeting IFIT2. Furthermore, immunoprecipitates significantly enriched in the anti-AGO2 group of hSCAP overexpressing miR-199a-5p were highly correlated with the type-1 IFN signaling pathway. Taken together, these findings suggest that increasing miR-199a-5p expression promotes bone regeneration during AP, which may be partly through the regulation of IFIT2 expression and type-1 IFN signaling (**Figure 7**). Thus, miR-199a-5p may be potentially utilized as a therapeutic target to facilitate bone defect repair in AP and to be identified as a diagnostic maker for AP.

## Data availability statement

The datasets presented in this study can be found in online repositories. The names of the repository/repositories and accession number(s) can be found below: the miRNA and mRNA sequencing data are available in the SRA database with the BioProject ID PRJNA931286 and PRJNA930996, respectively. Our RIP-seq data can be accessed in the SRA database with the BioProject ID PRJNA933173. 

## Ethics statement

The studies involving human participants were reviewed and approved by the Ethics Committee of The Affiliated Hospital of Stomatology, Chongqing Medical University. Written informed consent to participate in this study was provided by the participants’ legal guardian/next of kin. The animal study was reviewed and approved by the Ethics Committee of The Affiliated Hospital of Stomatology, Chongqing Medical University.

## Author contributions

JH: Study design, conducting experiments, data acquisition and collation, figure drawing, and manuscript draft. XH: Sample collection and data analysis. LZ and YZ: Data acquisition and analysis. HuZ and LN: Data analysis and collation. XP and HoZ contributed to the conception, design, and data interpretation of the study and critically revised the manuscript. All authors provided final approval and agree to be accountable for the content of the work.
