# Supplementary material for: MiR-199a-5P promotes osteogenic differentiation of human stem cells from apical papilla via targeting IFIT2 in apical periodontitis
Source: Front Immunol. 2023 Mar 30;14:1149339. doi: 10.3389/fimmu.2023.1149339 (PMC10098181; doi:10.3389/fimmu.2023.1149339)
Supplement: Supplementary file 1 [file DataSheet_1.pdf]

Supplementary Material

1 Supplementary Figures

1.1 Figure S1

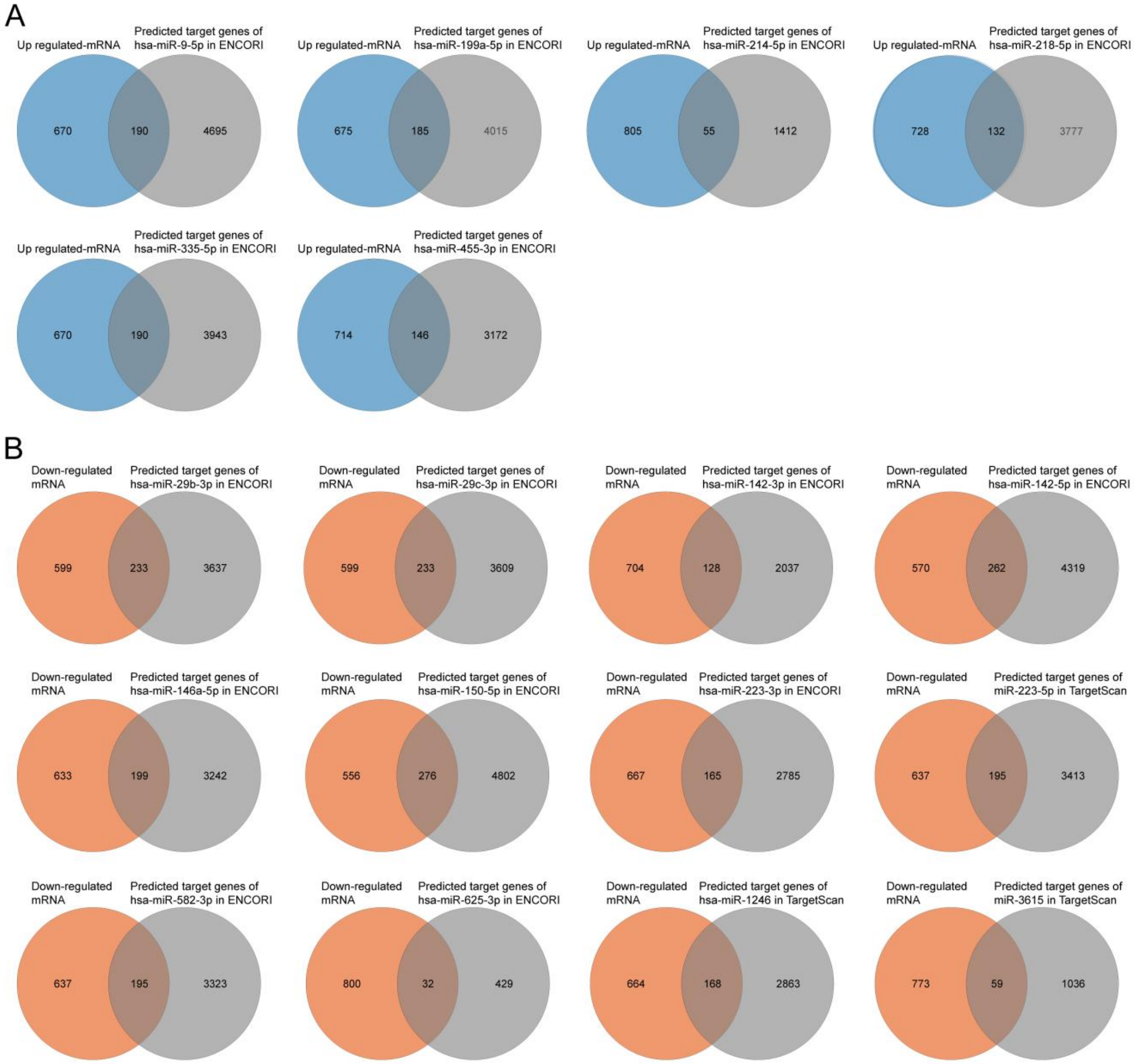

Figure S1. Intersection genes of the DE-mRNAs and the predicted target genes of DE-miRNAs. **(A)** The up-regulated mRNAs intersected with the predicted target genes of 6 down-regulated miRNAs. (**B**) The down-regulated mRNAs intersected with the predicted target genes of 12 up-regulated miRNAs.

1.2 Figure S2

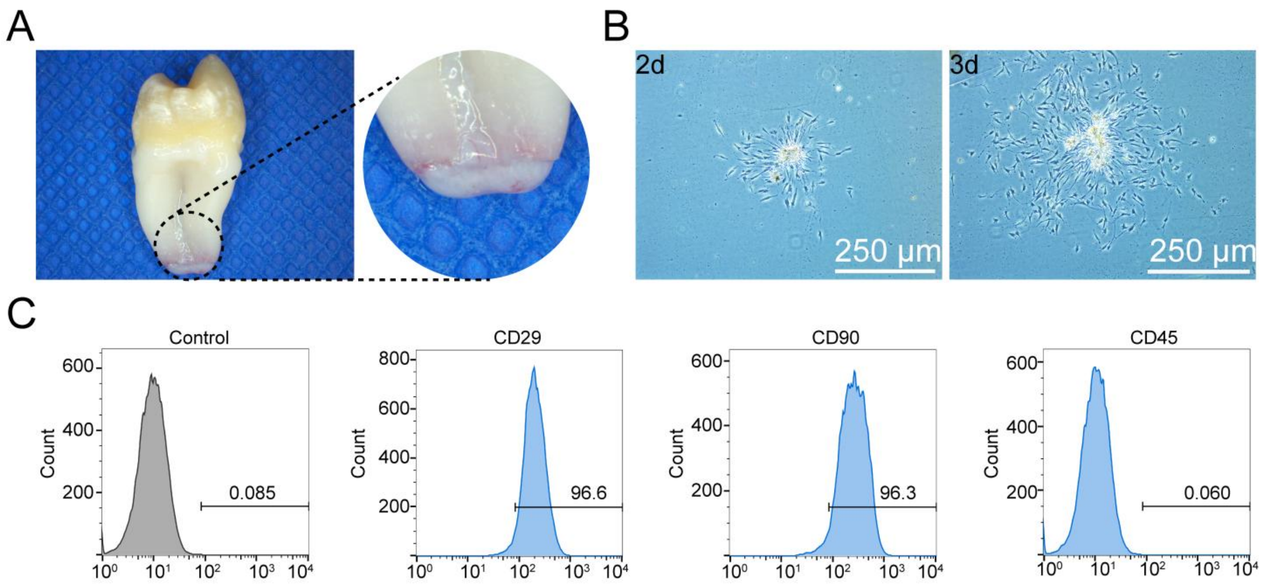

Figure S2. Isolation and identification of hSCAPs. (A) The apical papilla of immature young permanent teeth (dotted circles). (**B)** Primary hSCAPs on days 2 and 3. (**C)** Flow cytometric analysis of hSCAPs. Percentage values showed their positive expression patterns.

1.3 Figure S3

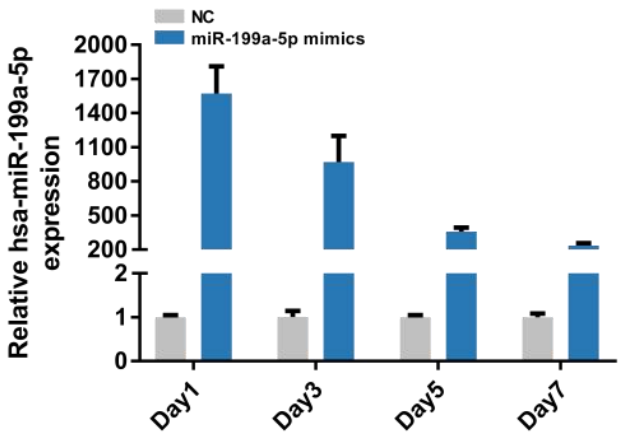

Figure S3. The relative expression level of miR-199a-5p after transfection of miR-199a-5p mimics into hSCAPs in vitro for several days.

1.4 Figure S4

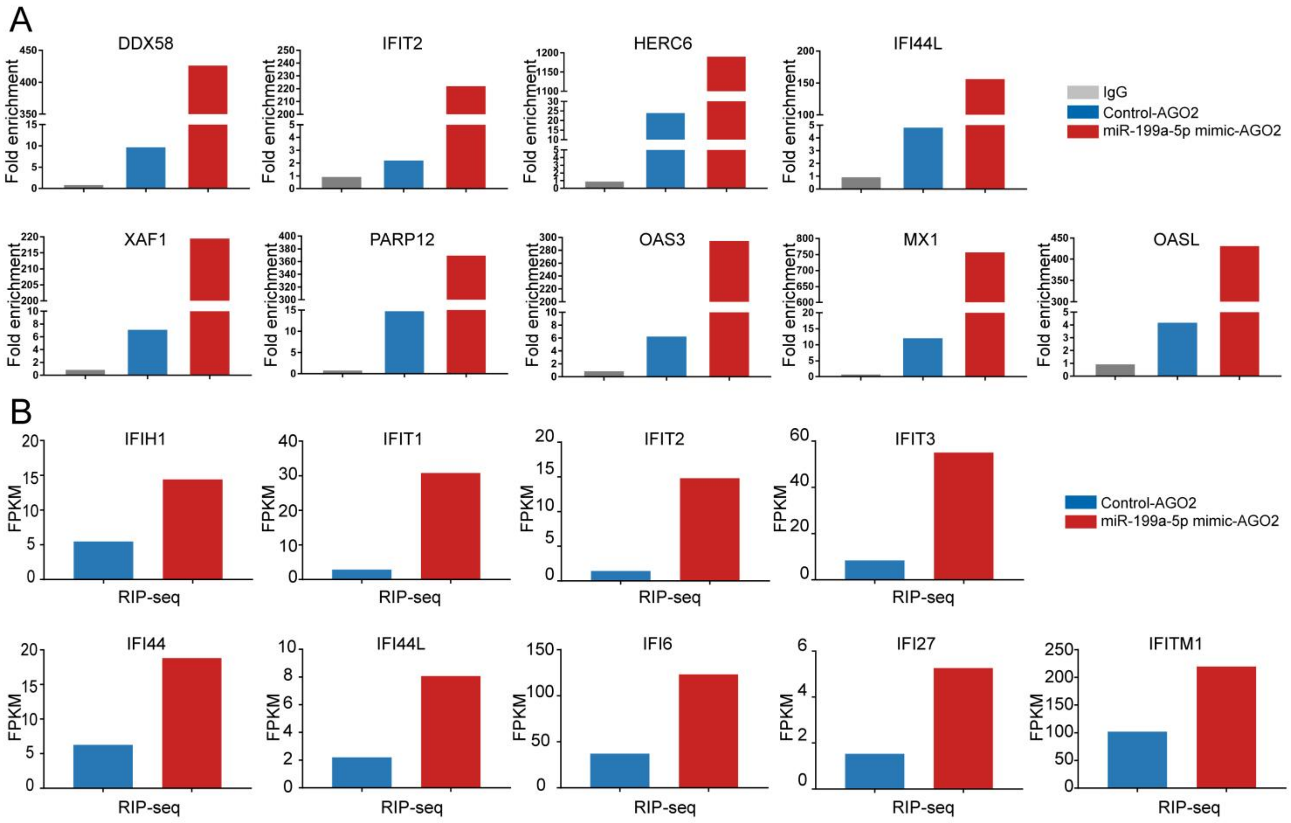

Figure S4. The relative expression level of 9 potential target genes and ISG family genes in RIP products. (A) The relative expression level of 9 potential target genes in anti-AGO2 immunoprecipitates between the miR-199a-5p overexpression group and the control group by qPCR. **(B)** ISG family genes including IFIT1, IFIT2, IFIT3, and IFI44L enriched in anti-AGO2 immunoprecipitates.

1.5 Figure S5

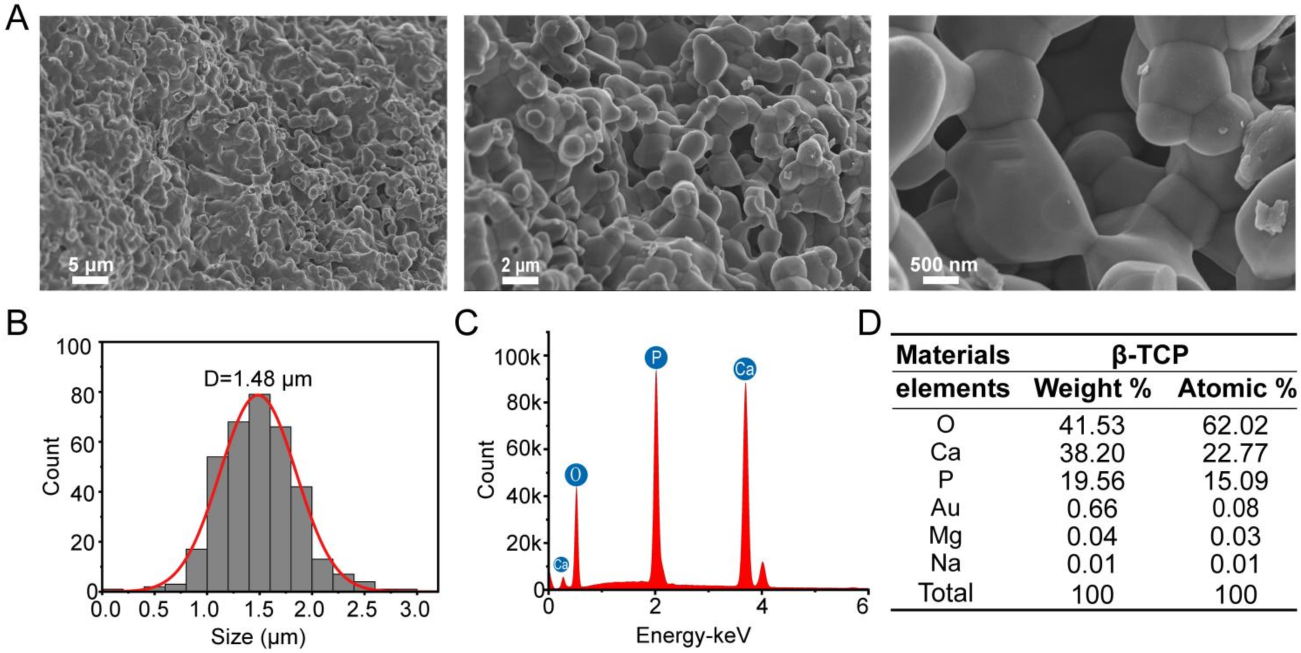

Materials	β-TCP	
elements	Weight %	Atomic %
O	41.53	62.02
Ca	38.20	22.77
P	19.56	15.09
Au	0.66	0.08
Mg	0.04	0.03
Na	0.01	0.01
Total	100	100

Figure S5. The physical and chemical properties of *β*-TCP ceramic particles. (A) The SEM images of *β*-TCP ceramic particles. **(B)** Analysis of particle size distribution. **(C, D)** EDS analysis of the scaffolds.

1.6 Figure S6

A

Variable	Frequency	Percent	Mean
Age (yrs) (mean±SD)			7
Patient (healthy control/AP)	36(13/23)	36.1/63.9	
Gender (M/F)	14/22	38.9/61.1	
Apical periodontitis (PAI score:3/4/5)	4/8/11	17.4/34.8/47.8	
healthy control (PAI score:1/2)	10/3	76.9/23.1	

B

PAI Score	Bone alterations in mineral structures
1	Normal periapical structures
2	Small changes in bone structures
3	Change in bone structure with mineral loss
4	Periodontitis with well-defined radiolucent area
5	Severe periodontitis with exacerbating features

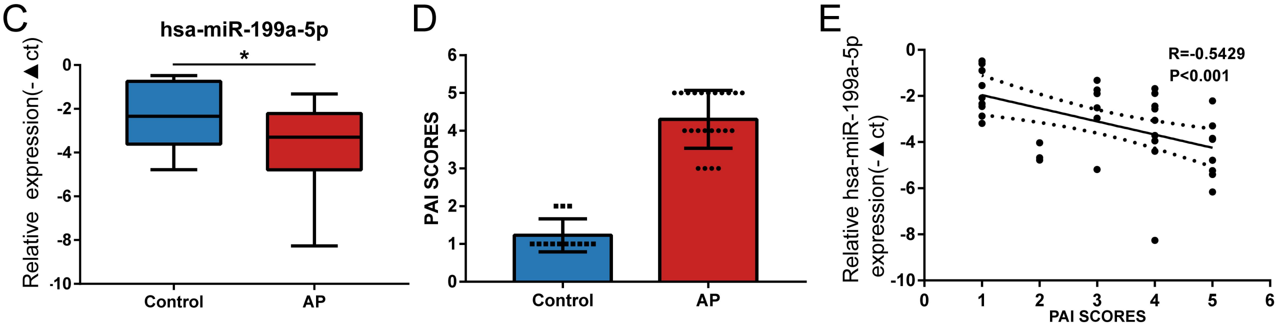

Figure S6. (A) The characteristics of the patients.**(B)** periapical index (PAI) scoring system (Venskutonis, 2016). **(C)** The relative expression of miR-199a-5p in the healthy control and AP groups. **(D)** The PAI scores in the healthy control and AP samplesgroups. **(E)** Spearman correlation analyses between the PAI scores and miR-199a-5p expression in periapical tissue samples ($n = 36$). Data were presented as the mean ± SD. $*p < 0.05$.

2. Supplementary Tables

2.1 Table S1. Inclusion and exclusion criteria

	inclusions criteria	exclusions criteria
Control	1. Children aged 6-8 years whose permanent anterior teeth have erupted and the corresponding deciduous teeth have not yet fallen out (i.e., Retained deciduous teeth)<br>2. Retained anterior deciduous teeth without caries<br>3. The remaining root length of the retained anterior deciduous teeth is greater than 2/3.	1. Children older than 8 years old or younger than 6 years old.<br>2. Children with systemic diseases: e.g. heart disease, asthma, diabetes, etc.<br>3. Retained anterior teeth with caries.<br>4. Root resorption of anterior teeth greater than 1/3.
AP	1. Children aged 6-8 years whose deciduous molar teeth have not yet undergone physiological roots resorption.<br>2. Indications for extraction of deciduous molars with severe periapical periodontitis:<br>a) teeth with inflammatory root resorption and ineffective root canal treatment;<br>b) Periapical inflammation spreading to the underlying permanent tooth germ.	1. Children older than 8 years old or younger than 6 years old.<br>2. Children with systemic diseases: e.g. heart disease, asthma,diabetes, etc.<br>3. Teeth with apical periodontitis of non-carious origins.

2.2 Table S2. sequences of hsa-miR-199a-5p mimics and antagomir

Gene	sequence(5'-3')
hsa-miR-199a-5p-mimics-ss	CCCAGUGUUCAGACUACCUGUUC
hsa-miR-199a-5p-mimics-as	ACAGGUAGUCUGAACACUGGGUU
hsa-miR-199a-5p-antagomir	ACAGGUAGUCUGAACACUGGGUU

2.3 Table S3. List of the primers used in the study

Gene	Sequence	Use
GAPDH	Forward GTCTCCTCTGACTTCAACAGCG<br>Reverse ACCACCCTGTTGCTGTAGCCAA	qRT-PCR
RUNX2	Forward CCCAGTATGAGAGTAGGTGTCC<br>Reverse GGGTAAGACTGGTCATAGGACC	
OSX	Forward TTCTGCGGCAAGAGGTTCACTC<br>Reverse GTGTTTGCTCAGGTGGTCGCTT	
ALP	Forward GCTGTAAGGACATCGCCTACCA<br>Reverse CCTGGCTTTCTCGTCACTCTCA	
OCN	Forward CGCTACCTGTATCAATGGCTGG<br>Reverse CTCCTGAAAGCCGATGTGGTCA	
OPN	Forward CGAGGTGATAGTGTGGTTTATGG<br>Reverse GCACCATTCAACTCCTCGCTTTC	
IFIT2	Forward GGAGCAGATTCTGAGGCTTTGC<br>Reverse GGATGAGGCTTCCAGACTCCAA	
MX1	Forward GGCTGTTTACCAGACTCCGACA<br>Reverse CACAAAGCCTGGCAGCTCTCTA	
OASL	Forward GAGTGGAAGGAAGAGGTGCTAGA<br>Reverse TCCATATCAGCCTCAGAACATCTT	
IFI44L	Forward TGCACTGAGGCAGATGCTGCG<br>Reverse TCATTGCGGCACACCAGTACAG	
DDX58	Forward CACCTCAGTTGCTGATGAAGGC<br>Reverse GTCAGAAGGAAGCACTTGCTACC	
HERC6	Forward GGAGAAACGACTTCCATTGATGTG<br>Reverse AGCTCTGAGCAGATCATCCTCG	
XAF1	Forward CCTCCATGAGGCTTACTGCCTG<br>Reverse GAAACTCCAGCGAGGACTTCTG	
OAS3	Forward CCTGATTCTGCTGGTGAAGCAC<br>Reverse TCCCAGGCAAAGATGGTGAGGA	
PARP12	Forward CTCTGTCACCAAACCTCCACAC<br>Reverse GCTACTGCTGACAGTGGTCACA	

2.4 Table S4. List of the primers used in the study

Gene	Sequence	Use
hsa-miR-199a-5p	Forward GCCCAGTGTTCAGACTACCTGTT	qRT-PCR (tailed)
hsa-miR-335-5p	Forward GGTCAAGAGCAATAACGAAAAATG	
hsa-miR-9-5p	Forward CGAGTCTTTGGTTATCTAGCTGTATG	
hsa-miR-218-5p	Forward CGAGTTGTGCTTGATCTAACCAT	
hsa-miR-455-3p	Forward GGCAGTCCATGGGCATATACA	
hsa-miR-29b-3p	Forward GAGTAGCACCATTTGAAATCAGTGT	
hsa-miR-223-3p	Forward GAGTGTCAGTTTGTCAAATACCCC	
U6	Forward CTCGCTTCGGCAGCACA<br>Reverse AACGCTTCACGAATTTGCGT	
hsa-miR-199a-5p	Forward GGCCCCAGTGTTCAGACTAC<br>Common-Reverse ACTGCAGGGTCCGAGGTATT	qRT-PCR (Stem-loop)
hsa-miR-199a-5p	GTCGTATCGACTGCAGGGTCCGAGGTATTCGCAGTCG ATACGACGAACAG	RT (Stem-loop)
siIFIT2-1	Forward CCUGGAAUGCUUACGUAAA<br>Reverse UUUACGUAAGCAUUCCAGG	siRNA silencing
siIFIT2-2	Forward GCCAGACAAAGCGAUUGAA<br>Reverse UUCAAUCGCUUUGUCUGGC	
siIFIT2-3	Forward CUCAGACGUUCAGAUUUAU<br>Reverse AUAAAUCUGAACGUCUGAG	

Reference

VENSKUTONIS, T. 2016. Periapical tissue evaluation: analysis of existing indexes and application of Periapical and Endodontic Status Scale (PESS) in clinical practice. *Giornale Italiano di Endodonzia,* 30**,** 14-21.
